# Hearty Suppers

**Published:** 1860-11

**Authors:** 


### HEARTY SUPPERS.

In this exaggerating age, we think we can safely say, that scarcely a day passes in which we do not receive, personally or by letter, some manifestation of felt indebtedness to the wholesome influence of this Journal; and if the question is asked, In what direction? it is most frequently answered, "In reference to the benefits derived from abstinence, in whole or in part, from eating any thing later than a mid-day dinner." It was with a feeling of painful disappointment, with perhaps some vexation, that we recently read of the death of a brother editor, whose excellent monthly seldom failed of some extract from, or kindly notice of, this Journal. He died in the very prime of life—not thirty-one—in the midst of usefulness, and in the enjoyment of usual good health, until within twenty-four hours of his decease. He was an able preacher, and a fine *belles-lettre* scholar. He was on a journey, on the Master's business, and died from home. He had made up the copy for his September issue. Two of the articles were from our August Number; one a plea for women, the other for children. So many good people loved him and looked up to him! In less than three lines the whole story is told. "He traveled all day, ate in the evening a hearty supper, waked up in the morning with a headache, became unconscious, and died at five o'clock in the afternoon, of apoplectic disease!"

Eating heartily in an exhausted, or even in a greatly debilitated bodily condition, is dangerous at any hour. Many a man has fallen apoplectic, at the close of a hearty dinner; but the danger is greatly increased by going to bed soon after; for the weight of the meal, a pound or two, rests steadily on the great veins of the body, arrests the flow of the blood, as a continuous pressure of the foot on a hose-pipe will more or less completely stop the flow of water along it. This arrestment causes a damming up of blood in the vessels of the brain, which at length can not longer bear the distension, and burst, causing effusion there, which is instant, sometimes, and certain death always.

There is scarcely a reader, of middle life, who has not more than once been nearer death than he imagined, from this very cause. A man feels in his sleep as if some terrible calamity was impending, some horrible beast is after him, or some fearful flood is about to overwhelm him; but, spite of every effort, he can not remove himself sufficiently fast; the enemy behind is increasing upon him; and at length, in an agony of sweat, he is able by a desperate effort, to set the stream of life in motion by uttering some sound, fearful to be heard, or only saves himself from falling into some fathomless abyss, by a convulsive and desperate effort. In cases where there is no power to cry out, or no effort can be made, the person is overtaken, or falls, and dies! Eating a hearty meal at the close of the day, is like giving a laboring man a full day's work to do, just as night sets in, although he has been toiling all day. The whole body is fatigued when night comes, the stomach takes its due share, and to eat heartily at supper, and then go to bed, is giving all the other portions and functions of the body repose, while the stomach has thrown upon it five hours more of additional labor, after having already worked four or five hours to dispose of breakfast, and a still longer time for dinner. This ten or twelve hours of almost incessant labor has nearly exhausted its power; it can not promptly digest another full meal, but labors at it for long hours together, like an exhausted galley-slave at a newly-imposed task. The result is, that by the unnatural length of time in which the food is kept in the stomach, and the imperfect manner in which the exhausted organ manages it, it becomes more or less acid; this generates wind; this distends the stomach; this presses itself up against the more yielding lungs, confining them to a largely diminished space; hence every breath taken is insufficient for the wants of the system, the blood becomes foul, black, and thick, refuses to flow, and the man dies, or in delirium or fright, leaps from a window or commits suicide, as did Hugh Miller, and multitudes of others, as to whom the coroner's jury has returned the non-committal verdict, "Died from causes unknown," if not more impiously stating, "Died by the visitation of God."

Let any reader who follows an inactive life for the most part, try the experiment for a week, of eating absolutely nothing after a two o'clock dinner, and see if a sounder sleep and a more vigorous appetite for breakfast and a hearty dinner, are not the pleasurable results, to say nothing of the happy deliverance from that disagreeable fullness, weight, oppression, or acidity, which attends over-eating. The greater renovation and vivacity which a long, delicious, and connected sleep imparts, both to mind and body, will of themselves more than compensate for the certainly short and rather dubious pleasure, of eating a supper with no special relish.

